# Association of socioeconomic status and health-related behavior with elderly health in China

**DOI:** 10.1371/journal.pone.0204237

**Published:** 2018-09-20

**Authors:** Fubaihui Wang, Qingkai Zhen, Kaigang Li, Xu Wen

**Affiliations:** 1 China Institute of Sport Science, Beijing, China; 2 Department of Exercise Science, College of Health and Human Science, Colorado State University, Fort Collins, Colorado, United States of America; 3 Department of Sport Science, College of Education, Zhejiang University, Hangzhou, China; Universidad del Desarrollo, CHILE

## Abstract

Previous health studies have focused on the correlation between socioeconomic status (SES) and health. We pooled data from the Chinese Longitudinal Healthy Longevity Survey (N = 9765) conducted in 2011, and examined the association of SES and health-related behavior with elderly health in China. The cumulative health disadvantage of the elderly caused by SES can be relieved by lifelong health-related behavior. In the same SES, the odds of self-rated health (SRH) as “good,” mini-mental state examination (MMSE) as “not impaired,” and activities of daily living (ADLs) as “not impaired” among the elderly who exercised regularly, were 46.9%, 28.6%, and 62.3% lower for the elderly who rarely exercised. The elderly who started doing regular exercise from 30 years old, achieved higher SRH, ADL, and MMSE scores to some extent. The health improvement advantage for the elderly who started doing regular exercises after 60 years old, was reduced. However, the odds of SRH as “good,” MMSE as “not impaired,” and ADLs as “not impaired” were still 3.4%, 12.5%, and 17.8%, respectively, higher than the respondents who never exercised. The health-related behaviors not only promote elderly health improvement, but its duration has also been found to be associated with the extent of health improvement.

## Introduction

The positive association between elderly health and socioeconomic status (SES) has been widely documented in the literature [1] and can be summarized into two perspectives: social causation and health selection [2]. Social causation proposes that the individual health level is associated with social structure position [3]. Individuals with higher SES possess greater health advantages than those with lower SES. A superior work and living environment can also reduce risks of health damage [4]. A high income increases the advantages of medical and service access, enabling individuals to cultivate a relatively healthy lifestyle [5, 6]. On the contrary, health selection deems that health condition is a screening mechanism of social mobility. Health gaps among different SES groups may be the result of “selection.” Individuals with better health conditions may gain higher SES, and individuals with poorer health conditions tend to move “downward” to lower social classes. These phenomena expand the health gradients among different classes, thereby generating health inequality [7, 8].

The health differential caused by SES has been appropriately described and summarized in empirical studies and review articles [1]. A controversy still exists over the relationships among SES, health-related behavior, and health. One view is that SES, rather than psychosocial factors or medical care, is the fundamental cause of health differences [9]. Another view is that SES and lifestyle preferences independently influence health [10,11,12]. For example, low SES directly affects health through lack of material resources and indirectly influences health by contributing to unhealthy behaviors, such as smoking [1]. The healthy difference accumulates continuously with increased age, finally leading to healthy cumulative disadvantages [13]. The effect of SES is gradually weakened, and the effect of biological factors is gradually enhanced and even exceeds SES of old age [14]. Thus, social relationship, social support, and control ability are reduced gradually until they disappear and no longer causing health difference [13,15].

Therefore, the elderly health in China cannot be explained on the basis of social causation or health selection. The purpose of the present study is to examine the association of SES and health-related behavior with elderly health in China. We explore whether health-related behavior is a mediator of the relation between SES and elderly health.

## Theoretical framework

The relation between health and SES has been observed in many countries [16]. The elderly health level in a country or region is affected by the prevailing social, political, and economic conditions [13]. China has a distinct political background and a different socioeconomic development course from developed countries [17]. Therefore, elderly health inequality in China should be understood through the local social situation. Economic development, demographic transition, and cultural integration essentially affect the life course of the Chinese [18]. The life course perspective holds that human development and aging are life-long processes. The SES of different life stages may be related to the elderly health; it is not generated occasionally or randomly, but is related to individual life experiences and behavior. A better understanding of the relationships among SES, individual healthy behavior, and health requires a long-term perspective that fully recognizes how SES and individual healthy behavior can influence one's health from birth until death [19].

### Elderly health level

A great number of academic researchers have analyzed how social disadvantage can result in poor health from the perspective of life course [20, 21]. A number of demographic, medical, and epidemiological studies have provided evidence of linkages between the SES of different life stages and health in later life [22,23]. Hayward and Gorman found that men's adult mortality is associated with their childhood SES [22]. Zeng Yi et al. showed that receiving adequate medical service during sickness in childhood significantly reduces the impaired risk of activities of daily living (ADL) and cognitively at the oldest-old ages [24]. They argued that the lower SES of childhood and adulthood influence the elderly health, explaining the relationship between SES and health at older ages [25]. The disadvantage of SES leads to health inequality, and health inequality in old age is the cumulative manifestation of lifelong health inequality.

### Socioeconomic status (SES)

SES is increasingly recognized to have long-term effects on health and mortality at older ages [26,27]. A number of studies have documented significant associations between early-life SES and elderly health [28,29]. The research method suggests that the SES indicators of childhood include birthplace (rural vs. urban) and childhood family economic status [30]. Moreover, the SES indicators of adulthood include residence (rural vs. urban) and family economic status during adulthood.

As part of the early-life conditions, the disease environment is believed to be worst in rural than in urban areas. Despite vast improvements in public health and sanitary campaigns in rural areas in the subsequent decades, the negative effects of sanitation on the early health of life still remain. The previous studies have shown that parental occupations and income are significantly associated with their children’s health in adulthood. For example, Gilman and colleagues (2002) found that children from low-income families had nearly a two-fold increase in the risk of major depression in adulthood compared with those from high-income families [31]. Beebe-Dimmer et al. (2004) found that childhood conditions in terms of father’s occupation and education are correlated with a 31-year mortality risk in adult women [32]. Thus, childhood family economic status is often measured by the primary occupation and education of the father before 60 years old [33], and the adulthood family economic status is often measured by the primary occupation, educational background, and financial situation of the respondent before reaching the age of 60 years old. Educational attainment reflects the opportunities for survival and health in early life [24]. In the early twentieth-century China, educational opportunities were limited and only a few people with good SES can pursue education. Education (i.e., any schooling) and income (i.e., economic independence and living conditions) are robust indicators of the SES resources available to the elderly in China [25]. Thus, respondents can receive more health benefits if they and their respective fathers pursued education [24].

### Health-related behavior

Health behavior is significant in improving the health of the elderly. Health behavior is a positive action by the elderly to prevent disease and maintain health; it includes changing dangerous lifestyles, reducing or eliminating health-risk behavior (e.g., smoking, alcohol, and unhealthy diet), taking active health behavior (e.g., regular exercise and regular physical examination), and complying with doctors’ recommendations. It is one of the most effective approaches to control non-communicable diseases and save medical expenses in the elderly.

Many scientific scales are available to measure health behavior and are often used in the study on elderly health. Smoking, exercise, and diet are associated with health and mortality [22]. Smoking and alcohol consumption are often selected to represent health damage behavior [6]. Regular exercise is also used to represent health promotion behavior. In the current study, we only used the three variables, namely, smoking, alcohol consumption, and regular exercise, to measure health-related behavior.

### Current study

The purpose of the present study is to examine the following hypotheses:

1. Childhood SES and adulthood SES are associated with elderly health, and such an association is higher for adulthood SES.
2. Health-related behavior mediates the relationship between SES and elderly health.
3. Active health-related behavior, specifically in early life, is associated with improving the elderly health status and reducing health problems in old age.

## Methods

### Data

The study used the data from the sixth wave of Chinese Longitudinal Healthy Longevity Survey (CLHLS) conducted in 2011. The CLHLS was initiated to satisfy the requirements of scientific research on the oldest-old. The survey was conducted in 1998, 2000, 2002, 2005, 2008, and 2011. It randomly selected half of the counties/cities in 23 Chinese provinces, the populations of which constitute approximately 85% of the total population in China [24]. The questionnaire was provided in a retrospective survey. The respondent information, such as age, gender, educational history, and family economic situations (including childhood, adulthood, and old age) were repeatedly asked in each wave. The CLHLS data were proven to be valid and reliable, according to a careful data quality evaluation (i.e., reliability coefficients, factor analysis, rates of logically inconsistent answers, and mortality follow-up) and assessment of the self-reported ages of participants [24,34,35]. The sixth wave survey covered a total of 10,191 samples. We eliminated the respondents younger than 60 years old and the samples with missing key variables. Finally, 9,765 effective respondents were retained (Table 1).

**Table 1:** Descriptive statistics for variables (unweighted).

Variable	Percentage	Variable	Percentage
**Health level**		Has quit smoking before 60 years old	4.34
*Activities of daily living (ADL)*		Never	66.16
Impaired	28.69	*Drink (always)*	12.52
Not impaired	71.31	Not previously drinking but presently drinking	4.49
*Mini-Mental State Examination (MMSE)*		Temperance after 60 years old	13.08
Impaired	62.46	Temperance before 60 years old	1.34
Not impaired	37.54	Never	68.57
*Self-rated health (SRH)*		*Regular Exercise (never)*	
Good	82.58	Started before 15	2.35
Not good	17.42	Started during 15–29	5.76
**SES variables during childhood**		Started during 30–44	1.43
*Primary occupation of father before 60 years old*		Started during 45–59	4.75
low-level occupations	86.03	Started after 60	31.06
high-level occupations	13.97	**Demographic variables**	
*Educational background of father*		*Gender*	
Educated	15.49	Male	45.04
Not educated	84.51	Female	54.96
*Birthplace*		*Nationality*	
Rural	89.13	Han	86.05
Urban	10.87	Minority	13.95
**SES variables during the adult stage**		*Year of Birth*	
*Education background*		1942–1951	18.21
Educated	41.7	1932–1941	25.55
Not educated	58.3	1922–1931	27.89
*Current place of residence*		Before 1921	23.92
Rural	52.69	*Residential form in adulthood*	
Urban	47.31	Living with family	79.92
*Primary occupation before 60 years old*		Not living with family	20.08
Low-level occupations	68.8	*Marital status in adulthood*	
High-level occupations	31.2	Married	37.87
*Current financial situation*		Single, widowed and divorced	62.13
Adequate source of income	79.69	*Medical service during the childhood*	
Inadequate source of income	20.31	Receives adequate medical services	35.28
**Health-related behaviors**		Not receives adequate medical services	64.72
*Smoking (always)*	15.55	*Frequent starvation during the childhood*	
Not previously smoking but presently smoking	2.38	Yes	75.71
Has quit smoking after 60 years old	11.57	No	24.29

The CLHLS study was approved by the research ethics committees of Peking University (IRB00001052-13074). All participants provided written informed consent. No experimental interventions were performed. All data were fully anonymized before the researchers accessed them.

### Measures

#### Elderly health

The elderly health was measured by the self-rated health (SRH), ADLs, and mini-mental state examination (MMSE). The three indicators were commonly used for measuring elderly health. The SRH is a common subjective health measurement index. Previous studies have shown that it is more accurate in measuring health and risk factors [36,37] and is often used to evaluate health conditions [2,17,38]. SRH in the questionnaire is divided into five levels, including very healthy, healthy, acceptable, unhealthy, and very unhealthy. The first three levels were integrated into “good,” and the remaining two levels were integrated into “poor”.

ADLs were converted into health variables, namely, impaired and not impaired, based on the previous measurement methods [39]. ADL impairment was considered when the respondent cannot independently fulfill at least one of the following six activities: taking a shower, dressing oneself, dining, toilet use, indoor activities, and defecation control [40]. MMSE was converted into two health variables: impaired and not impaired.

MMSE was used to measure the cognitive function of the elderly. The MMSE scale had a total of 30 scores and covered cognitive functions in terms of positioning ability, attention, calculation ability, memory ability, and language ability. MMSE impairment was recorded when the total score of respondent was less than 18 [41].

#### Childhood SES

Birthplace and family economic status were used to measure childhood SES. Birthplace was divided into rural and urban. The father’s occupation was classified based on previous research methods, where physical labor was considered low-level occupation and mental labor was considered high-level occupations [3,42]. This was coded as follows: high-level occupations, including professional, technical, governmental, institutional, and managerial occupation and industrial, commercial, and military occupation, and low-level occupations, including employment in agriculture, forestry, animal husbandry, fishing, and housework. Twenty percent of the fathers of respondents (≥60 years old) pursued education. The educational background of the fathers were coded into two categories, namely, educated or not.

#### Adulthood SES

The place of residence, educational background, primary occupation of respondents before 60 years old, and current financial situation were used to measure the adulthood SES. Educational background was divided into two categories, namely, educated or not because only 43% of the respondents pursued education in the data. The primary occupation before 60 years old was also divided into low and high levels. Income was used to measure the financial situation.

#### Health-related behavior

Two different measures of health-related behavior were used. First, we coded the variables of health-related behavior dichotomously. Smoking was answerable by “Yes” or “No,” according to whether a respondent smoked at the moment. Drinking and regular exercise were also answerable by “Yes” or “No.” Then, to test the cumulative effect, the variables of smoking, drinking, and regular exercise were re-encoded using multi-categorical measure, according to the time dimension. Smoking was classified as follows: never, quit smoking before 60 years old, quit smoking after 60 years old, not previously smoking but presently smoking, and always [4]. Similarly, drinking was classified into never, temperance before 60 years old, temperance after 60 years old, not previously drinking but presently drinking, and always. Regular exercise was recorded as regular exercise before 15 years old, regular exercise from 15–30 years old, regular exercise from 30–45 years old, regular exercise from 45–60 years, regular exercise after 60 years old, and never.

#### Demographic and other potential control variables

Participants reported age, gender, ethnicity, and family-related information. The information included family sanitary condition in childhood and family support in adulthood. Family sanitary condition in childhood was measured by the following two variables: whether the elderly received adequate medical services during childhood and whether they suffered frequent starvation during childhood [43]. The two factors were used to evaluate the relationship between SES and health status. Zeng et al. found that receiving adequate medical services during childhood illness was associated with a significantly lower risk of physical activity limitations, cognitive impairment, and self-reported poor health at ages 80 and above [25]. Some scholars have used the indicator of suffering frequent starvation in childhood to evaluate nutrition and health status in adulthood [23]. They believe that these health outcomes were highly significant predictors of mortality [44]. Family support in adulthood was measured by two variables: living with family (or not) and marital status.

### Statistical analysis

A logistic regression model was constructed through the stepwise addition of independent variables. The Stepwise regression method was used to solve collinearity problem among variables. After adding a new variable, a statistical test of each variable was added to the equation to test whether the independent variables that were degenerated into statistical insignificance should still be included. This test was repeated until no independent variable can be inputted nor eliminated, thereby obtaining an “optimal” regression equation. The analysis process is described below.

First, three logistic regression models were constructed to analyze the association between childhood SES and elderly health.

Second, variables of adulthood SES were added to examine associations with elderly health, childhood SES, and adulthood SES, and then to determine which had a greater positive relation on the elderly health.

Third, variables of health-related behavior were added to the model to examine whether health-related behavior mediated the relationship between SES and elderly health.

Finally, the variables of health-related behavior were divided by time sequence and then added to the model. We examined whether active health-related behavior, specifically that which started in early life, are associated with improving the elderly health status and reducing the health disadvantages in old age.

## Results

Table 1 shows that the percentage of SRH as “good” is 82.58% and the percentage of ADLs as “not impaired” is 71.31%. However, the percentage of MMSE as “not impaired” is only 37.53%, indicating that the mental health of the Chinese elderly is an important problem. For SES variables during childhood, 86.03% of the respondents were born in rural. Moreover, 86.03% of the respondents’ fathers are engaged in low-level occupation and 84.51% of the respondents’ fathers failed to pursue education. For SES variables during the adult stage, 58.3% of the respondents did not pursue education. The percentage of current “rural” residents is 52.69%, 68.8% of respondents are engaged in low-level occupations before 60 years old, and 79.69% of respondents have adequate sources of income.

### Relationship between SES and the elderly health

Females presented with significantly poorer health level than males. The odds of SRH as “good,” MMSE as “not impaired,” and ADLs as “not impaired” are 28.6%, 38.4%, and 16.4% lower for females than for males, respectively. Although the SRH and MMSE of minorities are relatively lower than the Han nationality, the odds of ADLs as “not impaired” is 1.762 times higher than those of Han nationality. Better MMSE and ADLs are achieved in subsequent birth cohorts.

In the SES variables of childhood, “education” is found to be statistically significant. The non-educated respondents are less healthy than the educated respondents in old age. The odds of MMSE as “not impaired” in non-educated respondents are reduced by 46.5%. Family economic status during childhood is significantly correlated with MMSE. If the respondents did not starve frequently during childhood, then the odds of MMSE as “not impaired” increased by 24.8%.

The relationship between adulthood SES and elderly health became more significant after adding the SES variables of adulthood. The MMSE and ADLs of married respondents are better than those of single, widowed, and divorced individuals. The respondents who engaged in high-level occupations before 60 years old showed better SRH, higher MMSE, and poorer ADLs than those engaged in low-level occupations. This finding may be explained by two aspects. One aspect is the theory of “natural selection”. The respondents who engaged in low-level occupations and survived in poverty possessed better health originally. Another aspect is that the judgment based on their low MMSE may be inaccurate. Income in old age is derived from the accumulation of SES during adulthood. The elderly who possessed an adequate income to satisfy daily requirements exhibited evident health advantages. The odds of SRH as “good,” MMSE as “not impaired,” and ADLs as “not impaired” are 71.8%, 32.1%, and 24% lower, respectively, for the elderly with inadequate incomes. In previous studies, some researchers agreed that most of the effects of childhood conditions on oldest-old mortality are indirect [2]. The findings are consistent with the literature. We also concluded that the relationship between adulthood SES and elderly health is stronger than childhood SES in terms of the significance level of variables or risk coefficient (Tables 2 and 3).

**Table 2:** Relationship between childhood SES and the elderly health.

Dependent variables	SRH (Good)		MMSE (Not Impaired)		ADL (Not Impaired)	
	OR	SE	OR	SE	OR	SE
**Control variables**						
Year of birth (before 1921)						
1922–1931	0.729**	0.069	1.696***	0.149	2.352***	0.176
1932–1941	0.679***	0.065	3.259***	0.28	5.763***	0.497
1942–1951	0.753**	0.082	5.338***	0.503	9.72***	1.11
Minority(Han Nationality)	0.642***	0.08	0.509***	0.065	1.762***	0.257
Female (male)	0.714***	0.052	0.616***	0.038	0.836*	0.058
Did not receive medical services during childhood (yes)	1.118	0.078	0.950	0.057	0.996	0.067
Did not frequently starved during childhood (frequent)	1.023	0.079	1.248**	0.081	1.029	0.077
**SES variables in childhood**						
Birthplace: rural (urban)	0.949	0.108	0.812*	0.076	1.164	0.121
Occupation of father: high-level: (low level)	1.097	0.116	1.08	0.093	0.927	0.091
Educational background of father: not educated (educated)	1.108	0.098	0.91	0.067	0.878	0.078
Educational background: not educated (educated)	0.808**	0.065	0.535***	0.035	0.928	0.072
	LR chi2(11) = 76.00		LR chi2(11) = 1068.16		LR chi2(11) = 818.27	
	Log likelihood = –3089.4204		Log likelihood = –3852.8394		Log likelihood = –3300.812	
	Prob> chi2 = 0.0000		Prob> chi2 = 0.0000		Prob> chi2 = 0.0000	
	Pseudo R2 = 0.0122		Pseudo R2 = 0.1217		Pseudo R2 = 0.1103	

Note

*: p<0.05

**: p<0.01

***: p<0.001

**Table 3:** Relationship between SES and the elderly health.

Dependent variables	SRH (Good)		MMSE (Not Impaired)		ADL (Not Impaired)	
	OR	SE	OR	SE	OR	SE
**Control variables**						
Year of birth (before 1921)						
1922–1931	0.792*	0.078	1.683***	0.150	2.279***	0.174
1932–1941	0.766*	0.080	3.06***	0.279	5.417***	0.510
1942–1951	0.899	0.109	4.926***	0.506	9.038***	1.115
Minority (Han Nationality)	0.699**	0.091	0.532***	0.069	1.801***	0.265
Female (male)	0.674***	0.054	0.67***	0.043	0.832*	0.062
Marital status: single, widowed, divorced (married)	1.132	0.095	0.783***	0.053	0.821*	0.065
Residential form: not living with family (living with family)	0.974	0.088	1.071	0.082	1.781***	0.155
Did not frequently starved during childhood (frequent)	0.974	0.078	1.209**	0.079	1.033	0.078
Did not receive medical services during childhood (yes)	1.149	0.083	0.958	0.058	0.988	0.067
**SES variables during childhood**						
Birthplace: rural (urban)	1.027	0.125	0.898	0.088	1.039	0.113
Occupation of father: high-level: (low level)	0.989	0.109	0.963	0.086	0.957	0.097
Educational background of father: not educated (educated)	1.08	0.098	0.901	0.067	0.866	0.077
Educational background: not educated (educated)	0.897	0.076	0.585***	0.039	0.877	0.07
**SES variables during adulthood**						
Current place of residence: urban (rural)	1.057*	0.074	1.035	0.062	0.882	0.058
Occupation before 60 years old: high-level (low level)	1.099*	0.107	1.334***	0.1	0.761**	0.066
Financial situation: inadequate (adequate)	0.282***	0.020	0.679***	0.047	0.76***	0.056
	LR chi2(11) = 403.66		LR chi2(11) = 1135.48		LR chi2(11) = 885.92	
	Log likelihood = –2915.7767		Log likelihood = –3806.6302		Log likelihood = –3257.0942	
	Prob> chi2 = 0.0000		Prob> chi2 = 0.0000		Prob> chi2 = 0.0000	
	Pseudo R2 = 0.0647		Pseudo R2 = 0.1298		Pseudo R2 = 0.1197	

Note:

*: p<0.05

**: p<0.01

***: p<0.001

### Association of SES and health-related behavior and their impact on elderly health

The relationship between SES and the elderly health is altered after adding the health-related behavior variables. ADLs are unrelated to gender, and SRH is unrelated to education. Health-related behavior i significantly associated with the elderly health. As a health promotion behavior, “regular exercise” showed significant associations with the elderly health. In the same SES, for the elderly who exercised rarely, the odds of SRH as “good,” MMSE as “not impaired,” and ADLs as “not impaired” are 46.9%, 28.6%, and 62.3% lower, respectively, than the elderly who exercised regularly (Table 4).

**Table 4:** Association of SES and health-related behavior on the elderly health.

Dependent variables	SRH (Good)		MMSE (Not Impaired)		ADL (Not Impaired)	
	OR	SE	OR	SE	OR	SE
**Control variables**						
Year of birth (before 1921)						
1922–1931	0.744**	0.075	1.611***	0.146	2.143***	0.169
1932–1941	0.674***	0.072	2.872***	0.267	4.819***	0.457
1942–1951	0.753*	0.094	4.523***	0.475	7.804***	0.991
Minority (Han Nationality)	0.704**	0.093	0.529***	0.07	1.834***	0.275
Female (male)	0.796**	0.068	0.705***	0.049	0.962	0.077
Marital status: single, widowed, divorced (married)	1.132	0.096	0.771***	0.053	0.809*	0.066
Residential form: not live with family(live with family)	0.946	0.087	1.084	0.084	1.851***	0.166
Did not receive medical services during childhood (yes)	1.16*	0.086	0.968	0.059	1.018	0.072
Did not frequently starved during childhood (frequent)	1.007	0.082	1.228**	0.081	1.07	0.083
**SES variables during childhood**						
Birthplace: rural (urban)	1.008	0.125	0.879	0.087	1.084	0.121
Occupation of father: high-level: (low level)	0.98	0.11	0.955	0.087	0.953	0.1
Educational background of father: not educated (educated)	1.088	0.101	0.897	0.068	0.888	0.082
Educational background: not educated (educated)	0.929	0.08	0.601***	0.041	0.919	0.076
**SES variables during adulthood**						
Current place of residence: urban (rural)	1.004	0.072	0.998	0.061	0.829**	0.056
Occupation before 60 years old: high-level (low level)	1.021	0.102	1.265**	0.097	0.672***	0.061
Financial situation: inadequate (adequate)	0.28***	0.020	0.682***	0.048	0.781**	0.062
**Health-related behavior**						
Smoking: No (Yes)	0.784*	0.079	0.971	0.074	0.811*	0.076
Drinking: No (Yes)	0.563***	0.060	0.749***	0.056	0.695***	0.065
Regular exercise: No (Yes)	0.531***	0.041	0.714***	0.042	0.377***	0.027
	LR chi2(11) = 515.95		LR chi2(11) = 1165.81		LR chi2(11) = 1095.34	
	Log likelihood = –2811.9172		Log likelihood = –3710.2561		Log likelihood = –3081.5294	
	Prob> chi2 = 0.0000		Prob> chi2 = 0.0000		Prob> chi2 = 0.0000	
	Pseudo R2 = 0.0840		Pseudo R2 = 0.13588		Pseudo R2 = 0.1509	

Note

*: p<0.05

**: p<0.01

***: p<0.001

Theory of life course suggests that life-long health-related behaviors have a cumulative effect on elderly health. We analyzed the relationship between health-related behavior duration and the improvement of elderly health. The variables of health-related behavior were divided by time sequence and added to the model. Results indicate that the significant levels of adulthood SES variables are altered to some extent. The relation of residence and primary occupation before 60 years old with the elderly health is smaller, but the relationship between income and elderly health is more significant.

The model result show that the respondents with long-term habits of smoking and drinking have better SRH and ADLs than those who never or have quit such unhealthy habits. Compared with the respondents who always drink, the odds of SRH as “good” and ADLs as “not impaired” are 61.4% and 58.2% lower for those who never smoked, and 29.4% and 39.3% lower for those who quit drinking before 60 years old, respectively. Some researchers have also reported similar conclusions. They confirmed that unhealthy behavior influenced the death rate to a lower extent among the elderly than among middle-aged individuals and also explained natural selection theory, which postulates that only the elderly with better health can continue to maintain unhealthy lifestyles [45]. However, we found that the MMSE impairment of the elderly with unhealthy behavior is higher. The elderly with unhealthy behavior are more likely to obtain higher odds of MMSE impairment. The odds of MMSE as “not impaired” are 14.7% higher for those who never drank and 12.4% higher for those who quit drinking before 60 years old, than for respondents who have always been drinking.

The elderly, who started regular exercises earlier in life have evident health advantages and higher health levels than those of the other groups. The odds of SRH as “good,” ADLs as “not impaired,” and MMSE as “not impaired” are 118.4%, 48.3%, and 81.9% higher for those who started regular exercise at 30–44 years old, respectively, than respondents who never exercised. Thus, starting a healthy lifestyle in the middle age can effectively improve elderly health levels. Although the health improvement advantage for the aged who started exercises after 60 years old is reduced, the odds of SRH as “good,” MMSE as “not impaired,” and ADLs as “not impaired” are still 3.4%, 12.5%, and 17.8% higher, respectively, than the respondents who never exercised (Table 5).

**Table 5:** Association of duration of health-related behavior on the elderly health.

Dependent variables	SRH (Good)		MMSE (Not Impaired)		ADL (Not Impaired)	
	OR	SE	OR	SE	OR	SE
**Control variables**						
Year of birth (before 1921)						
1922–1931	0.784*	0.085	1.733***	0.174	2.234***	0.19
1932–1941	0.715**	0.083	3.108***	0.323	5.124***	0.529
1942–1951	0.872	0.12	5.258***	0.626	1.287***	1.489
Minority (Han Nationality)	0.625**	0.092	0.524***	0.081	1.887***	0.314
Female (male)	0.694**	0.074	0.731***	0.064	0.79*	0.076
Marital status: single, widowed, divorced (married)	1.057	0.101	0.776**	0.062	0.827*	0.074
*Residential form*: not live with family(live with family)	0.999	0.102	1.113	0.098	1.959***	0.195
Did not receive medical services during childhood (yes)	1.153	0.095	1.035	0.073	0.982	0.076
Did not frequently starved during childhood (frequent)	0.989	0.09	1.242**	0.094	1.054	0.089
**SES variables during childhood**						
Birthplace: rural (urban)	0.958	0.129	0.874	0.096	0.996	0.12
Occupation of father: high-level: (low level)	0.858	0.105	0.956	0.097	0.901	0.102
Educational background of father: not educated (educated)	1.03	0.107	0.939	0.081	0.88	0.088
Educational background: not educated (educated)	0.948	0.092	0.557***	0.044	0.882	0.08
**SES variables during adult stage**						
Current place of residence: urban (rural)	1.026	0.083	0.896	0.064	0.812**	0.061
Occupation before 60 years old: high-level (low level)	1.104	0.125	1.258*	0.114	0.748**	0.076
*Financial situation*: inadequate (adequate)	0.268***	0.021	0.647***	0.052	0.709***	0.059
**Health-related behavior**						
Smoking (always)						
Previously not smoking but presently smoking	1.238	0.216	1.032*	0.215	1.508	0.328
Has quit before 60	0.684*	0.101	1.121*	0.009	0.795	0.157
Has quit after 60	0.844	0.013	1.045*	0.048	0.21**	0.085
Never	0.349	0.111	1.691**	0.018	0.873	0.108
Drinking (always)						
Previously not drinking but presently drinking	1.169	0.22	1.008	0.171	1.343	0.18
Has quit before 60	0.706***	0.073	1.124*	0.013	0.607**	0.092
Has quit after 60	0.801***	0.025	1.253	0.371	0.937**	0.132
Never	0.386***	0.085	1.147**	0.008	0.418**	0.086
Regular Exercise (never)						
Started before 15	1.417	0.258	1.513*	0.164	1.942***	0.348
Started during 15–29	1.154	0.181	1.402**	0.179	1.203	0.176
Started during 30–44	2.184*	0.797	1.483*	0.118	1.819*	0.545
Started during 45–59	1.129	0.119	1.301*	0.306	1.175	0.127
Started after 60	1.034**	0.21	1.125***	0.159	1.178*	0.143
	LR chi2(11) = 398.17		LR chi2(11) = 945.79		LR chi2(11) = 841.92	
	Log likelihood = –2274.0446		Log likelihood = –2945.79		Log likelihood = –2568.6822	
	Prob> chi2 = 0.0000		Prob> chi2 = 0.0000		Prob> chi2 = 0.0000	
	Pseudo R2 = 0.0805		Pseudo R2 = 0.1420		Pseudo R2 = 0.1408	

Note

*: p<0.05

**: p<0.01

***: p<0.001

## Discussion

One's SES in both childhood and adulthood are associated with the elderly health. We find that the relationship between adulthood SES and the elderly health is more significant than childhood SES. The occupation level, financial situation, and residential conditions are significantly associated with the elderly health. These findings support the previous research conclusions stating that health condition is positively correlated with SES [46].

Different opinions exist on whether health differences among the elderly with different SES are expanding or narrowing in China. We believe that the relationship between SES and the elderly health can be explained as follows. First, high SES can reduce health risks by improving the living environments and forming healthy lifestyles, thereby improving the elderly health level. Second, the respondents who survived poverty and other difficulties may have underwent natural selection. They originally have stronger physical health than others. However, we also find that although the elderly with long-term habits of smoking and drinking have better SRH and ADL scores than those who never or have quit smoking and drinking, the odds of MMSE impairment remains high.

We also find that health-related behavior are significantly associated with the elderly heath, and lifelong regular exercises can relieve the cumulative disadvantages of elderly health caused by SES. The relationship between SES and elderly health is weakened after the health-related behavior is added in the model, thereby indicating that health-related behavior mediates the relationship between SES and the elderly health. This finding can be explained from the following two aspects. First, people with high SES have stronger motivation to perform health-related behaviors. Second, individuals with high SES have better abilities to maintain healthy lifestyles, especially financial support, when they are young. The health-related behavior can reduce health risks throughout life, thereby improving the health level in old age.

The elderly health disadvantage caused by SES can be mediated by health-related behavior. Active health-related behavior, specifically starting early in life, is significantly associated with improving the elderly health status and reducing the health disadvantages in old age. The health-related behavior cannot only promote elderly health improvement, but its duration can also be related to the extent of health improvement. Performing health-related behaviors from the middle age can improve SRH, ADLs, and MMSE significantly. Furthermore, the cumulative health disadvantage at the old-age stage caused by SES can be relieved by lifelong health-related behavior.

This study also has limitations. The data used only covered the respondents who were alive at the time of the six-wave investigation in 2011, but excluded the elderly who already died before this year. The death information, which may exhibit completely different health features from the respondents surveyed, remains unknown. Thus, the conclusions may only apply to the elderly, who survived after “natural selection”.

## Conclusions

Childhood SES and adulthood SES are associated with elderly health; the association is higher for adulthood SES. The cumulative health disadvantage in the old-age stage caused by SES can be mediated by health-related behaviors. Such behaviors not only promote elderly health improvement, but its duration also affects the extent of health improvement. Active health-related behavior in early life is associated with improving elderly health and reducing the health disadvantages in old age.
